# Effect of polymorphisms in the *Slc11a1* coding region on resistance to brucellosis by macrophages *in vitro* and after challenge in two *Bos* breeds (Blanco Orejinegro and Zebu)

**DOI:** 10.1590/S1415-47572010000300014

**Published:** 2010-09-01

**Authors:** Rodrigo Martínez, Susana Dunner, Rubén Toro, Jaime Tobón, Jaime Gallego, Javier Cañón

**Affiliations:** 1 Grupo de Recursos Genéticos y Biotecnología Animal, Centro de Biotecnología y Bioindustria, Corporación Colombiana de Investigación Agropecuaria, C.I., Bogotá DCColombia; 2 Departamento de Producción Animal, Facultad de Veterinaria, Universidad Complutense de Madrid, MadridSpain; 3 Centro de Investigación CEISA, Bogotá DCColombia; 4 Grupo de Recursos Genéticos y Biotecnología Animal, C.I. El Nus, San Roque, AntioquiaColombia

**Keywords:** Blanco Orejinegro Creole breed, Brahman breed, *Brucella abortus*, genetic resistance, zebu

## Abstract

The resistance/susceptibility of selected cattle breeds to brucellosis was evaluated in an F1 population generated by crossing animals classified as resistant (R) and susceptible (S) (R x R, R x S, S x R, S x S) based on challenges *in vitro* and *in vivo*. The association between single nucleotide polymorphisms identified in the coding region of the *Slc11a1* gene and resistance/susceptibility was estimated. The trait *resistance or susceptibility to brucellosis*, evaluated by a challenge *in vitro*, showed a high heritable component in terms of additive genetic variance (h^2^ = 0.54 ± 0.11). In addition, there was a significant association (p < 0.05) between the control of bacterial survival and two polymorphisms (a 3'UTR and SNP4 located in exon 10). The antibody response of animals classified as resistant to infection by *Brucella abortus* differed significantly (p < 0.05) from that of susceptible animals. However, there was no significant association between single nucleotide polymorphisms located in the *Slc11a1* gene and the antibody response stimulated by a challenge *in vivo*.

## Introduction

Resistance to disease is a particularly important attribute of livestock in low input production systems in the tropics and developing countries and is often the critical factor in the sustainability of such systems. Before starting a genetic improvement program it is important to demonstrate 1) that genetic improvement for disease resistance is an effective, low risk method, 2) that there is enough genetic variation for disease resistance between and within breeds, and 3) that there will be clear economic and social benefits resulting from a genetic improvement in resistance; the fulfillment of these three criteria allows the use of a range of alternative methods for disease control (Gibson and Bishop, 2005).

Although genetic variation is an important factor in conferring resistance or tolerance to a wide range of pathologies, the source of such variation, *i.e.*, through resistance to infection, tolerance of infection or a combination of both, remains unclear. Gibson and Bishop (2005) reviewed more than 50 diseases for which there is strong evidence of genetic variation in host resistance or tolerance, including most domestic livestock species.

Significant genetic variability for resistance/susceptibility to brucellosis has been detected in cattle (Feng *et al.*, 1996; Barthel *et al.*, 2001) and buffalo (Borriello *et al.*, 2006; Capparelli *et al.*, 2007a,b). In cattle, part of this resistance has been associated with a 3' untranslated polymorphism in the *Slc11a1* gene (microsatellite 3'UTR) (GT)n (Adams and Templeton, 1998; Horin *et al.*, 1999; Barthel *et al.*, 2001) that apparently does not affect the function of Nramp1 protein. The *Slc11a1* gene has received little attention, although Ables *et al.* (2002) described polymorphisms in introns 4 and 5 and exon V. More recently, Coussens *et al.* (2004) described the structural organization of this gene and identified polymorphisms in intron 10, although these were not associated with functional mutations related to resistance to brucellosis.

Brucellosis in cattle is caused by *Brucella abortus* and is characterized clinically by abortion during the last three months of pregnancy. Consequently, this disease is an important cause of economic losses (Meador *et al.*, 1989) and has a potentially high zoonotic risk (Ashford *et al.*, 2004). However, since abortion is not a pathognomonic sign of brucellosis (Cunningham, 1977) the estimated abortion rate in an animal group may mask the true extent of infection by *B. abortus* and therefore cannot be interpreted as a measure of resistance or susceptibility. Conversely, 2%-9% of infections occur in a latent, persistent form (Rodríguez and Crespo, 2002).

In the last 60 years, vaccination has been the main method for controlling brucellosis. Although strain 19 of *B. abortus* is an effective vaccine in cattle, the use of this strain has some problems, including the fact that it is also infectious to humans (Meyer, 1985), that the protection obtained is not absolute and is challenge-dependent, and that serological tests cannot always distinguish between infected animals and animals with antibodies as a consequence of vaccination (Moriyon *et al.*, 2004). Since this strain also produces abortion in vaccinated cows (~1%, depending on the stage of pregnancy) and genital lesions in sires, a challenge *in vivo* with strain 19 may be used as an indicator of resistance-susceptibility to brucellosis.

The objective of this study was to evaluate brucellosis resistance in resistant (R) or susceptible (S) individuals of two cattle breeds (a Colombian Creole breed and a Brahman zebu breed) and their diallelic crosses (R x R, R x S, S x R, S x S), following a challenge with *B. abortus* strain 19 *in vitro* or *in vivo*. We also estimated the effect of polymorphisms in the coding region of the *Slc11a1* gene on resistance or tolerance to brucellosis.

## Materials and Methods

###  Animal population

Two hundred and seventy-five animals of two breeds (Blanco Orejinegro Creole or BON, n = 228, and Brahman or Zebu, n = 47) were used to assess bacterial survival *in vitro*. The sires and dams were classified as resistant (R) or susceptible (S) based on the survival of bacteria in cultured macrophages infected with *B. abortus*. Animals were mated in a diallelic cross design and macrophages from the progeny were used for evaluations *in vitro*.

For each group, four sires were mated to 95 cows and 70 progeny calves were used to assess bacterial survival *in vitro*. In addition, 40 paternal half-sibs from the Brahman breed were evaluated using *in vivo* and *in vitro* assays.

###  Monocyte-macrophage culture and infection *in vitro*

Monocyte-macrophages were cultured according to Price *et al.* (1990) and Qureshi *et al.* (1996). *Brucella abortus* Cumbal 1 strain (isolated from a field case) from the CORPOICA germplasm bank (Colombia) was maintained at 37 °C in a microanaerobiosis chamber (Oxoid, Hampshire, England) for four days in selective medium for *Brucella* (Oxoid) supplemented with 5% horse serum (Gibco BRL) and 5% dextrose (Sigma Chemical Co.). Bacteria were opsonized and used for macrophage infection as described elsewhere (Martínez *et al.*, 2008b). The macrophage monolayers (5 x 10^4^ macrophages) were challenged (in triplicate) with bacteria (~5 x 10^5^) at a multiplicity of infection (MOI) ratio of 10:1 in RPMI1640 medium supplemented with 15% heat inactivated fetal calf serum. RPMI-streptomycin medium (final concentration: 13.5 mg/mL) was added to eliminate extracellular bacteria before a further incubation at 37 °C for 30 min. The medium was subsequently aspirated from the wells and 200 μL of RPMI medium was added. Ten minutes later, 100 μL of this medium was skimmed off (to remove any residual streptomycin and dead bacteria) and another 100 μL of RPMI medium supplemented with 5% autologous heat-inactivated serum (from the same animal) was added.

To obtain results for time zero (T0h), the RPMI medium was immediately removed from the wells and 100 μL of deionized sterile cold water was added for 10 min. Aliquots of this deionized sterile cold water were used to prepare 1:5, 1:10 and 1:50 dilutions, and 100 μL of each dilution then plated (in triplicate) on a petri dish containing agar selective for *B. abortus* (Oxoid). The Petri dishes were incubated in a 5% CO_2_ atmosphere at 37 °C. The same serial dilutions were made at T24h and 100 μL aliquots again plated as described above. Colony forming units (CFUs) were counted four days later and referred to as the ‘number of bacteria at time zero' (NBT0) and ‘number of bacteria at time 24 h' (NBT24). Bacterial survival (rSOB24h) was estimated as the square root of the number of CFUs at 24 h (NBT24) relative to the number of CFUs at time zero (NBT0):



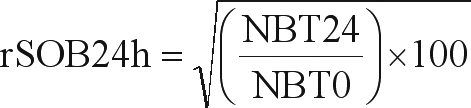


Based on the bacterial survival *in vitro* the animals were classified as having either a restrictive phenotype, *i.e.*, those in which macrophages were able to control bacterial survival (rSOB24h < 10), or a permissive/susceptible phenotype, *i.e.*, those that allowed bacterial replication and in which bacterial survival at 24 h was greater than at 0 h (rSOB24h > 10).

The experimental approach used here was based on a study by Price *et al.* (1990) who compared the results of an *in vitro* killing assay with those of an *in vivo* challenge: in the former assay, the results were reported as the percentage survival, with 100% of survival being used as the cut-off point to define restrictive phenotypes (resistant individuals). With this cut-off, the animal designation based on macrophage function was strongly correlated (r = 0.82) with the post-challenge phenotypic classification. However, Qureshi *et al.* (1996) used a 70% cut-off value that correlated perfectly with the number of animals allocated to each category and evaluated by an *in vivo* challenge.

###  Experimental challenge *in vivo*

In this experiment, 30 non-vaccinated animals (10 Zebu and 20 BON) of both sexes and similar age (18-30 months) and reproductive stage were used. The BON animals were divided into two groups (permissive n = 10 and restrictive n = 10) based on the ability of their macrophages to control bacterial survival *in vitro*. Throughout the experiment, the animals received a balanced diet of grass, sugar cane, corn silage and a mineral mixture. All of the animals were serologically negative for brucellosis before the challenge, as assessed by using a competitive ELISA.

The animals were challenged by the conjunctival administration of *B. abortus* strain S19. Each animal was inoculated in both eyes with 250 μL (500 μL/animal) of a bacterial suspension containing 6.0 x 10^9^ CFU/mL and then continuously monitored until the end of the experiment. The location and experimental protocol were approved by local sanitary authorities (ICA, Colombia).

Throughout the experiment, two blood samples were obtained periodically (at 0, 15, 30, 60 and 90 days post-infection) for immunological and bacteriological analyses. The serum samples were screened for anti-*B. abortus* antibodies with a competitive ELISA using a Svanovir *Brucella abortus* ELISA-c kit (Svanova 158, Biotech AB, Uppsala, Sweden), according to the manufacturer's recommendations. Serum samples and controls were assayed in duplicate, with the optical density being measured at 450 nm. The animals were classified as positive or infected when the percent inhibition (PI) was ≥ 40%.

Monthly lymph node biopsies were obtained by fine needle aspiration and the samples were processed and plated in petri dishes (in triplicate) containing agar selective for *B. abortus* (Oxoid). The Petri dishes were incubated in a 5% CO_2_ atmosphere at 37 °C and scored for CFUs after seven days.

###  Genotyping of *Slc11a1* polymorphisms

Four polymorphisms in the *Slc11a1* gene, including three SNPs (p.D321N – SNP4, p.P356A – SNP5, p.Q542del – SNP6) described by Martínez *et al.* (2008a) and a 3'UTR microsatellite described by Horin *et al.* (1999), were genotyped in all of the samples (Table 1). The DNA samples were subjected to PCR amplification using *Taq* DNA polymerase (Biotools, Madrid, Spain) and the following conditions: an initial incubation at 94 °C for 5 min, followed by 33 cycles of 95 °C for 1 min, 56 °C for 1 min and 72 °C for 1 min and finally 4 min at 72 °C. To detect the SNPs, 20-100 ng of each amplicon (with or without prior digestion by restriction enzymes, depending on its size) was subjected to single strand conformation polymorphism (SSCP) analysis by mixing the amplicon (v/v) with denaturing 2X SSCP loading buffer (95% formamide, 0.6% bromophenol blue and 0.6% xylene cyanole), followed by heating at 94 °C for 5 min, snap chilling on ice and loading onto 8% polyacrylamide gels prepared with TBE buffer. After electrophoresis (4 W, 15 h) at 10 °C, the amplicon bands were detected by silver staining (Bassam *et al.* 1991, Barroso *et al.*, 1997).

###  Statistical analysis

The variance components and heritability of the rSOB24h trait in the BON breed were calculated from the records of 228 animals (male progeny of 47 sires and 145 dams) that had data for this trait. The software DFREML (Meyer, 1988) was used to solve the following model:

*Y = X*β *+ Za + Zm + e*

where *Y* is the vector for observations (rSOB24h), β is the vector for fixed effects (breed, classification and sex), *X* is the incidence matrix for fixed effects, *a* is the vector for random additive genetic effects, *m* is the vector for random maternal effects, *Z* is the incidence matrix for random effects and *e* the vector for residual values. The non-parametric Kruskal-Wallis test was used to examine possible associations between SNP polymorphisms and the rSOB24h trait. Statistical comparisons were done using the SAS software version 8.1 (SAS Inst. Inc, Cary, Nc, USA, 2000).

Antibody titers were analyzed with a mixed model for repeated measures that included breed, classification (permissive or restrictive) and genotype as fixed effects and the animal as a random effect. The model used to analyze the antibody titers of anti-*B. abortus* is described by the relationship:

*Y = X*β *+ Za + e*

where *Y* is the vector for observations (anti-*B. abortus* antibody titers), β is the vector for fixed effects considered, *i.e.*, breed, resistance classification (permissive or restrictive), animal genotype and time post-infection (15, 30, 60 and 90 days), *X* is the incidence matrix for fixed effects, *a* is a vector for random effects (animal), *Z* is the incidence matrix for random effects, and *e* is the vector for residual values. The mixed procedure implemented in SAS software was used to solve the model.

## Results

###  Mating of resistant and susceptible animals

To identify the genetic component responsible for resistance/susceptibility to brucellosis in cattle, the phenotype of animals able to modulate bacterial survival (rSOB24h) was initially determined. This was done by evaluating macrophages obtained from BON animals selected from sires and cows classified as resistant (R) or susceptible (S), and then mating these animals to obtain the crosses R x R, R x S, S x R and S x S. The progenies of these crosses were used to study the genetic control of this trait.

The progeny of R x R, R x S and S x R crosses generally had a higher percentage of resistant animals (38%-44%) than the progeny of S x S crosses (25%); however, 75% of the animals in the latter cross showed greater bacterial survival (rSOB24h) than in the other crosses (Table 2). In several cases, there were significant differences in the number of resistant and susceptible animals among crosses.

**Figure 1:**
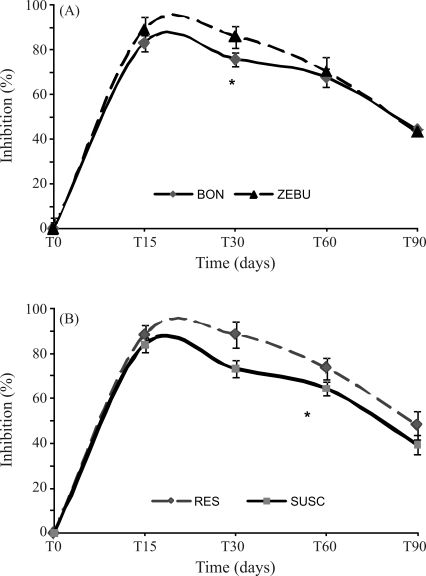
Influence of breed (A) and susceptibility to infection (B) on the anti-*B. abortus* antibody titers in sera of experimentally infected animals. The points are the mean ± SD of IP (Inhibition Percentage), R = resistant, S = susceptible. The curves in panel A contain the combined results for resistant and susceptible animals for each breed, and whether the curves for resistant and susceptible animals in the right-hand panel contain both BON and Zebu animals.

There was no difference in the bacterial survival *in vitro* between resistant animals of the two breeds, whereas a significantly greater survival was observed in susceptible Zebu animals compared to the corresponding BON animals (Table 3). Bacterial survival *in vitro* and the number of bacteria at time 0 (NBT0) showed moderate to high additive and maternal heritabilities (Table 4).

### *In vivo* challenge with *B. abortus* strain S19

The response to an *in vivo* challenge with *B. abortus* strain S19 was monitored by quantifying the anti-*B. abortus* antibody titers and the results were expressed as the percent inhibition (PI) of the antibody response. The antibody titer decreased from 83.2 at 15 days post-infection to 42.2 at 90 days post-infection, when 50% of the animals had a PI lower than the threshold (40%), indicating that animals were resistant to infection. Resistant animals showed significantly greater inhibition (p < 0.05) of the antibody response at 30 days and 60 days post-infection compared to susceptible animals (Figure 1). The differences between the two breeds were significant (p < 0.05) only during the first 30 days post-infection, when Zebu animals showed greater inhibition.

Zebu animals had significant higher anti*-B. abortus* antibody titers than BON animals at 30 days post-infection, but not were significant at 15 or 60 days when the titers were similar. When comparing the resistant *vs.* susceptible animals found significant higher titers were found in resistant animals than susceptible oness at 30 and 60 days, but not at 90 days when the difference were lower (Figure 1).

The only pathological effects of the bacteria were ocular alterations seen during the first week after infection; there were no abortions in females or genital effects in males. Bacteria were isolated from sub-scapular lymph nodes in 16% of the samples (5 animals). The bacterial isolates were obtained from three susceptible (mean rSOB24h = 17.0 ± 2.9) BON animals from the S x S cross and two susceptible (mean rSOB24h = 13.6 ± 5.7) Zebu animals; there were no significant differences in the antibody titers of these five animals.

###  Influence of *Slc11a1* polymorphisms on bacterial growth *in vitro*

SNP4 had a significant (p < 0.04) effect on bacterial survival *in vitro*, with heterozygotes having lower values, *i.e.*, a better control of bacterial survival *in vitro*. Conversely, the most frequent (0.59) homozygous genotype (GG) showed higher values of *in vitro* bacterial growth control (Table 5). Also, the 3'UTR microsatellite showed a significant effect (p < 0.05) on the trait variability, displaying the BB genotype higher values than the other genotypes. There was no association between bacterial survival and polymorphisms SNP5 and SNP6.

###  Influence of *Slc11a1* polymorphisms on anti-*B. abortus* antibody titers

Although none of the genetic variants located in the *Slc11a1* coding region significantly affected the antibody titers to *B. abortus*, individuals with GG for SNP4 generally had low antibody titers (63.0 ± 10.1) whereas AA (68.4 ± 6.2) and heterozygous (67.1 ± 5.0) individuals had higher values.

## Discussion

The control of brucellosis is currently based on serologic diagnosis, vaccination, the slaughter of infected animals and permanent sanitary control. Some of the characteristics of brucellosis, such as its long incubation time, asymptomatic carriers and limited vaccine protection, have made eradication difficult, particularly in the tropics. One approach to overcome this problem is selective breeding for genetic resistance based on the natural variability found in cattle.

The SLC11A1 gene has been implicated in the modulation of certain diseases (Blackwell and Searle, 1999; Blackwell *et al.*, 2000), and is associated with resistance to brucellosis in buffalo (Borriello *et al.*, 2006, Capparelli *et al.*, 2007a,b). In this study, we examined the relationship between bacterial survival (rSOB24h) and animal phenotype by crossing individuals resistant or susceptible to brucellosis and then examining the resistance of their progeny to infection.

There was no difference in the rSOB24h of the two populations, although susceptible Zebu showed greater bacterial survival; the latter effect probably reflected the reduced ability to control the intracellular growth of *B. abortus* rather than differences between resistant animals of both breeds. This conclusion was supported by the significant association between *Slc11a1*-3'UTR variant and susceptibility but not with resistance. Similar results were reported for buffalo by Borrielo *et al.* (2006), but this was not confirmed by Paixão *et al.* (2007). As shown here, heterozygous individuals had lower levels of bacterial survival; Zebu animals with the *Slc11a1*-3'UTR polymorphism BB had higher bacterial survival than the other genotypes, which suggests that the BB genotype predisposes to greater susceptibility.

The R X R crossbreed resulted in a moderate number of resistant individuals whereas the S X S crossbreed yielded a high number of susceptible animals, as already described by others (Templeton *et al.*, 1990; Adams and Templeton, 1998). As observed here, more than 40% of the descendants of resistant sires were also resistant, indicating a high level of heritability. This finding agrees with Templeton *et al.* (1990) who were able to increase the frequency of non-vaccinated resistant animals (from 18% to 54%) by selecting resistant sires. The high heritability (h^2^ > 0.50) for NBT0 and rSOB24h (bacterial survival *in vitro*) may indicate that few genes are involved in the expression of these traits, one of them possibly being SLC11A1, as suggested by Barthel *et al.* (2001).

The animals challenged with *B. abortus* had anti-*B. abortus* antibody titers > 80% at 15 days post-infection. These high antibody titers were maintained for 4-5 weeks and then declined slowly so that at 12 weeks post-infection ~45% of the animals had PI values > 40%. Discrepancies in the antibody titers compared to other reports (Aguirre *et al.*, 2002) may be partially explained by differences in the initial bacterial load, although there was no difference in the response between resistant and susceptible animals.

Bacteria were isolated from lymph node samples in only 16% of the animals, which included 33% of animals classified as susceptible. The only clear pathological symptom of this challenge was ocular irritation. Although *B. abortus* S19 can produce abortion in vaccinated and non-vaccinated adult cows and cause genital problems in adult bulls (Corner and Alton, 1981; Nicoletti *et al.*, 1990; Chin, 2000; Moriyon *et al.*, 2004), these effects were not observed here, possibly because this strain is not particularly pathogenic in the cattle breeds studied.

In contrast to our findings, Xavier *et al.* (2009) reported that the most common overt lesions in cattle infected experimentally with *B. abortus* strain 2308 were fibrous necrotizing placentitis in cows and fibrous pleuritis and peritonitis in fetuses. Microscopically, the most frequent alteration in infected cows was necrotic neutrophilic placentitis with a perivascular infiltrate that was associated with large numbers of *B. abortus* located intracellularly in macrophages and trophoblasts and extracellularly in necrotic tissues. Similar lesions were not observed here because of the low infective capacity of the S19 strain and because legal restrictions precluded the use of a pathogenic strain.

###  Influence of *Slc11a1* SNPs on bacterial growth *in vitro*

Animals heterozygous for the SNP4 marker had a greater capacity to control bacterial growth *in vitro*, whereas no such association was observed for the polymorphisms SNP5 and SNP6. SNP4 also has a significant effect on expression of the cytokine TNF-α in macrophages stimulated with *B. abortusin vitro* (Martínez R and colleagues, unpublished observations). TNF-α is pro-inflammatory and triggers apoptosis in infected cells by activating caspases (Male, 2003), this being the principal defense mechanism against brucellosis in cattle (Wyckoff, 2002).

Finally, the 3'UTR polymorphism had a significant effect on bacterial survival *in vitro*, with the GT_13_/GT_13_ genotype being associated with a high susceptibility to brucellosis, as also observed by Horin *et al.* (1999) and Barthel *et al.* (2001) for transfected RAW264.7 macrophages. A significant effect of this microsatellite on the susceptibility to brucellosis has also been observed in other species, such as buffalo (*Bubalus bubalis*) (Borriello *et al.*, 2006), in which monocytes from animals with the BB genotype (GT_36_/GT_36_) had a significantly greater ability to control the intracellular replication of several *Brucella* species *in vitro* (Caparelli *et al.*, 2007a,b), although Paixão *et al.* (2007) observed no correlation between the BB (GT_13_/GT_13_) genotype and antibody titers to *B. abortus* in cattle.

In conclusion, we have identified an important genetic component of resistance/susceptibility to brucellosis that could be useful in implementing a traditional selection program for brucellosis resistance. However, the use of molecular markers to assist such selection schemes requires additional research since the gene analyzed here showed only a weak association with resistance/susceptibility to brucellosis. Assessment of the effect of SNP4 on protein function and of the factors that contribute to different anti-*B. abortus* antibody responses in resistant and susceptible animals would be of great interest.

## Figures and Tables

**Table 1:** Primers used to detect SNPs in the coding region of the bovine *Slc11a1* gene.

SNP	Primer	Sequence	Fragment size (bp)
SNP4 (p.D321N)*	SNP4F	GGCTTGGAGGTCTGATTTTC	176
	SNP4R	CGTTGGCTTGCTTACTCCTT	
SNP5 (p.P356A)*	SNP5F	CAAGGAGTAAGCAAGCCAAC	350
	SNP5R	GCTGCCTTAAGGATCAAGGA	
3'UTR (GT)_10_/(GT)_12_	3'UTR-F	ATGGAACTCACGTTGGCTG	175
	3'UTR-R	AAGGCAGCAAGACAGACAGG	
SNP6 (p.Q542del)*	SNP6-F	TTCCTGTATGGGCTTCCTG	158
	SNP6-R	CTTGCTGCCTTCACACACAT	

*Amino acid location in the protein.

**Table 2:** Number and percentage of resistant animals and the extent of intracellular growth of *B. abortusin vitro* after 24 h (rSOB24h) for each cross indicated.

Mating cross	Number of animals	%	rSOB24h
R X R	8	38.1	7.72 ± 2.89
R X R	13	62.9	20.78 ± 9.32
R X S	6	42.9	5.79 ± 3.23
R X S	8	57.1	22.71 ± 6.75
S X R	4	44.4	6.70 ± 3.26
S X R	5	66.6	16.91 ± 4.40
S X S	6	25.0	8.84 ± 1.56
S X S	18	75.0	22.64 ± 9.79

The values are the mean ± SD. R = resistant, S = susceptible.

**Table 3:** Intracellular growth of *B. abortusin vitro* after 24 h (rSOB24h) in resistant and susceptible breeds of cattle (BON: Blanco Orejinegro Creole; Zebu: Brahman).

Breed	Classification	n	rSOB24h
BON	Resistant	85	5.39 ± 1.83
Zebu	Resistant	10	6.55 ± 5.77
BON	Susceptible	143	26.03 ± 1.56
Zebu	Susceptible	37	36.01 ± 4.88*

The values are the mean ± SD. *p < 0.05 compared to susceptible BON or Zebu resistant animals.

**Table 4:** Genetic parameters for the number of bacteria at time 0 (NBT0) and for intracellular growth *in vitro* after 24 h (rSOB24h) in the Blanco Orejinegro Creole breed.

Parameter	NBT0	rSOB24h
σ^2^_a_	7634619	322
σ^2^_m_	1987036	87
σ^2^_e_	1855721	138
σ^2^_p_	11477377	548
h^2^_d_	0.56 ± 0.03	0.58 ± 0.11
h^2^_m_	0.17 ± 0.08	0.16 ± 0.096

σ^2^_a_ = additive genetic variance, σ^2^_m_ = maternal variance, σ^2^_e_ = error variance, σ^2^_p_ = phenotypic variance, h^2^_d_ = direct heritability, h^2^_m_ = maternal heritability.

**Table 5:** Effect of *Slc11a1* polymorphism on bacterial survival *in vitro*.

Polymorphism	Genotype frequency (n)*	rSOB24h	p
SNP4			
GG	0.11 (9)	21.1 ± 4.1**	0.0408
AG	0.30 (23)	15.2 ± 4.8	
AA	0.59 (47)	17.1 ± 4.2	
SNP5			
GG	0.12 (9)	20.0 ± 6.6	0.4205
CG	0.31 (23)	16.5 ± 4.0	
CC	0.57 (43)	16.5 ± 4.9	
3'UTR			
BB	0.09 (21)	58.3 ± 7.2	0.01
AB	0.12 (26)	16.5 ± 4.7	
AA	0.77 (165)	18.5 ± 1.8	
SNP6			
158/164	0.04 (3)	14.7	0.5985
156/158	0.13 (10)	13.3	
158/158	0.66 (50)	17.5	
156/164	0.02 (2)	13.6	
156/156	0.01 (1)	6.5	
154/154	0.01 (1)	16.3	
158/160	0.01 (1)	11.5	
154/156	0.02 (1)	30.7	
160/164	0.01 (1)	9.6	
160/162	0.01 (1)	17.7	
154/158	0.02 (2)	22.9	
156/160	0.01 (1)	11.5	

*Number of animals, **Mean ± SEM.
